# Clinical Characterization of the Three Waves of COVID-19 Occurring in Southern Italy: Results of a Multicenter Cohort Study

**DOI:** 10.3390/ijerph192316003

**Published:** 2022-11-30

**Authors:** Mariantonietta Pisaturo, Antonio Russo, Viraj Pattapola, Roberta Astorri, Paolo Maggi, Fabio Giuliano Numis, Ivan Gentile, Vincenzo Sangiovanni, Annamaria Rossomando, Valeria Gentile, Giosuele Calabria, Raffaella Pisapia, Alessio Vinicio Codella, Alfonso Masullo, Valentina Iodice, Giancarlo Giolitto, Roberto Parrella, Giuseppina Dell’Aquila, Michele Gambardella, Felicia Di Perna, Nicola Coppola

**Affiliations:** 1 Infectious Diseases, Department of Mental Health and Public Medicine, University of Campania “L. Vanvitelli”, 80138 Napoli, Italy; 2 Infectious Diseases Unit, A.O. S Anna e S Sebastiano Caserta, 81100 Caserta, Italy; 3 Emergency Unit, PO Santa Maria delle Grazie, 80078 Pozzuoli, Italy; 4 Infectious Disease Unit, University Federico II, 80138 Naples, Italy; 5 Third Infectious Diseases Unit, AORN dei Colli, PO Cotugno, 80131 Naples, Italy; 6 IV Infectious Disease Unit, AORN dei Coli, PO Cotugno, 80131 Naples, Italy; 7 Hepatic Infectious Disease Unit, AORN dei Colli, PO Cotugno, 80131 Naples, Italy; 8 IX Infectious Disease Unit, AORN dei Coli, PO Cotugno, 80131 Naples, Italy; 9 First Infectious Disease Unit, AORN dei Coli, PO Cotugno, 80131 Naples, Italy; 10 Infectious Diseease Unit, A.O. San Pio, PO Rummo, 82010 Benevento, Italy; 11 Infectious Disease Unit, A.O. San Giovanni di Dio e Ruggi D’Aragona, 84135 Salerno, Italy; 12 VIII Infectious Disease Unit, AORN dei Coli, PO Cotugno, 80131 Naples, Italy; 13 Infectious Disease Unit, Ospedale Maria S.S. Addolorata di Eboli, ASL Salerno, 84025 Eboli, Italy; 14 Respiratory Infectious Diseases Unit, AORN dei Colli, PO Cotugno, 80131 Naples, Italy; 15 Infectious Diseases Unit, AO Avellino, 83100 Avellino, Italy; 16 Infectious Diseease Unit, PO S. Luca, Vallo della Lucania, ASL Salerno, 84078 Vallo della Lucania, Italy; 17 Pneumology Unit, AORN Caserta, 81100 Caserta, Italy

**Keywords:** SARS-CoV-2, COVID-19, pandemic waves, clinical outcome, mortality

## Abstract

Aims: To characterize patients hospitalized for COVID-19 in the three waves in Southern Italy. Methods: We conducted a multicenter observational cohort study involving seventeen COVID-19 Units in Campania, southern Italy: All adult (≥18 years) patients, hospitalized with a diagnosis of SARS-CoV-2 infection from 28 February 2020 to 31 May 2021, were enrolled. Results: Two thousand and fifteen COVID-19 hospitalized patients were enrolled; 392 (19%) in the first wave, 917 (45%) in the second and 706 (35%) in the third wave. Patients showed a less severe clinical outcome in the first wave than in the second and third waves (73%, 65% and 72%, respectively; *p* = 0.003), but hospitalization expressed in days was longer in the first wave [Median (Q1–Q3): 17 (13–25) v.s. 14 (9–21) and 14 (9–19), respectively, *p* = 0.001)] and also mortality during hospitalization was higher in the first wave than in the second and third waves: 16.6% v.s. 11.3% and 6.5%, respectively (*p* = 0.0001). Multivariate analysis showed that older age [OR: 1.069, CI (1046–1092); *p* = 0.001], a worse Charlson comorbidity index [OR: 1042, CI (1233–1594; *p* = 0.0001] and enrolment during the first-wave [OR: 1.917, CI (1.054–3.485; *p* = 0.033] were predictors of mortality in hospitalized patients. Conclusions: Improved organization of the healthcare facilities and the increase in knowledge of clinical and therapeutic management have contributed to a trend in the reduction in mortality during the three waves of COVID-19.

## 1. Introduction

Coronavirus disease 2019 (COVID-19) is a disease caused by severe acute respiratory syndrome, Coronavirus-2 (SARS-CoV-2). For the first time, Wuhan Municipal Health Commission, Hubei Province, China, reported a cluster of cases of pneumonia and a novel coronavirus was eventually identified in January 2020 [1], highly contagious and quickly spread around the world.

COVID-19 most often causes respiratory symptoms that can feel much like a cold, flu, or pneumonia. In some cases, patients with COVID-19 can develop severe difficulty breathing, causing a need for hospitalization and intensive care [2]. The risk of severe disease increases with increasing age; also subjects with underlying medical conditions, including heart disease, diabetes, dementia, oncological or lung disease, have a greater risk of developing severe COVID-19 [3,4,5,6,7,8,9,10,11]. In fact, previous meta-analyses reported a higher mortality rate from COVID-19 in patients with comorbidities [12,13,14].

As of 12 September 2022, about 21 million patients have been hospitalized in Italy because of COVID-19. The worse data since the start of the pandemic was registered on 23 November 2020, when 34,697 individuals were being treated in hospitals for COVID-19-related reasons [15].

From December 2019, COVID-19 has spread throughout the world and has been described as occurring in several waves. The term “wave”, as used in infectious disease cycles, describes the rising and declining trends of infections over a prolonged period. In Italy, three waves of COVID-19 have occurred; the first wave started on 28 February 2020 to July 2020, the second wave occurred in Autumn 2020 and the third wave in Winter-Spring 2021 [14].

The aim of this study was to characterize the demographic and clinical characteristics of patients hospitalized for COVID-19 in the three waves in Campania, a region of southern Italy, analysing the CoviCamp cohort (COVID-19 Campania Cohort); specifically, the prevalence of deaths in the three waves was evaluated. This analysis may allow to evaluate also the impact of the clinical management and the healthcare organization in the outcome of COVID-19 itself.

## 2. Methods

We conducted a multicenter observational cohort study, named CoviCamp, involving seventeen COVID-19 Units in eight cities of the Campania region in southern Italy: Naples, Caserta, Salerno, Benevento, Avellino, Pozzuoli, Eboli and Vallo della Lucania. For research purposes, an electronic dataset was designed for the collection of demographics, clinical, hematobiochemical, virological and therapeutic data of the subjects hospitalized for COVID-19.

### 2.1. Study Population

All adult (≥18 years) patients, hospitalized with a diagnosis of SARS-CoV-2 infection confirmed by a positive reverse transcriptase-polymerase chain reaction (RT-PCR) on a naso-oropharyngeal swab, from 28 February 2020 to 15 September 2021, at one of the centers participating in the study and were enrolled in the CoviCamp cohort. Exclusion criteria included people aged <18 years, and lack of clinical data and/or informed consent. No study protocol or guidelines regarding the criteria of hospitalization were shared among the centers involved in the study and the patients were hospitalized following the decision of physicians of each center.

### 2.2. Data Collection

At admission the demographic, clinical, hematobiochemical, virological and therapeutic data of the subjects hospitalized for COVID-19 were collected in an electronic database shared by all 17 centers participating in the study. From this database we extrapolated the data for the present study.

### 2.3. Definition

The microbiological diagnosis of SARS-CoV-2 infection was defined as a positive RT-PCR test on a naso-oropharyngeal swab. Viral RNA was extracted from naso-oropharyngeal swab with QIAamp Viral RNA Kits (Qiagen GmbH, Hilden, Germany); the detection of SARS-CoV-2 was performed by RT-PCR test using Bosphore^®^ Novel Coronavirus (Anatolia Diagnostics and Biotechnology Products Inc., İstanbul, Turkey) Detection Kit V3, by primers designed on three viral regions: E, ORF1ab, and N regions.

We defined patients with non-severe SARS-CoV-2 infection if they were asymptomatic or experienced a mild infection and did not need oxygen (O_2_) therapy; we defined patients with a severe disease if they required O_2_ therapy; in this definition we included patients needing management in an intensive care unit (ICU) and/or high flow nasal cannula or invasive/non-invasive mechanical ventilation and including also patients who died

The patients were followed until SARS-CoV-2-RNA negativity at naso-oropharyngeal swab and/or discharged from hospital or died.

We considered three different periods of admission: the first included patients admitted from March 2020 to 31 August 2020; the second included all patients admitted from 1 September 2020 to 31 January 2021; the third included all patients admitted from 1 February 2021 to 31 May 2021.

### 2.4. Ethical issue

The study was approved by the Ethics Committee of the University of Campania L. Vanvitelli, Naples (n°10877/2020). All procedures performed in this study were in accordance with the ethical standards of the institutional and/or national research committee and with the 1964 Helsinki declaration and its later amendments, or comparable ethics standards. Informed consent was obtained from all participants included in the study.

### 2.5. Statistical Analysis

For the descriptive analysis, categorical variables were presented as absolute numbers and their relative frequencies. Continuous variables were summarized as mean and standard deviation if normally distributed or median and quartiles (Q1–Q3).

We performed a comparison of patients in the different waves using chi square for categorical variables and the ANOVA when the variable was normally distributed (age; days of enrolment after symptoms onset) or Kruskal-Wallis test when variable was not normally distributed (Charlson Comorbidity Index; length of hospitalization; haematobiochemical parameters) for continuous variables.

The variable included in analyses were: number of males (2015 valid data); Age (2015 valid data); days of enrolment after onset of symptoms (113 valid data); Charlson Comorbidity Index (1858 valid data); patients with arterial hypertension (2002 valid data); patients with cardiovascular disease (2003 valid data); patients diabetes (2002 valid data); patients with malignancy (2001 valid data); patients with chronic kidney disease (2001 valid data); patients with chronic obstructive pulmonary disease (2002 valid data); patients with hepatopathy (1995 valid data); number of asymptomatic subjects (1991 valid data); number of patients with fever (1949 valid data), cough (1945 valid data), dyspnea (1949 valid data), hypo-ageusia (1859 valid data), hypo-anosmia (1864 valid data), diarrhea (1886 valid data), cutaneous lesions (1802 valid data); clinical outcome of COVID-19 non severe and severe (2015 valid data); number of patients died during hospitalization (2015 valid data); white blood cell count (1544 valid data); INR(1427 valid data); AST (1510 valid data); ALT (1409 valid data); LDH (1451 valid data); creatinine (1515 valid data); total bilirubin (1401 valid data); PaO_2_/FiO_2_ (1397 valid data). We performed a comparison according to the clinical outcome (non-severe COVID-19, severe COVID-19 and death during hospitalization) using Pearson chi-square or Fisher’s exact test for categorical variables and Student’s *t*- or Mann-Whitney tests for continuous variables. We used Student’s *t*-test when variable was normally distributed (age; days of enrolment after symptoms onset) and Mann-Whitney test when variable was not normally distributed (Charlson Comorbidity Index; length of hospitalization; haematobiochemical parameters). We performed a comparison of patients who were discharged from hospital and those who died during hospitalization using a Pearson chi-square test or Fisher’s exact test for categorical variables, and Student’s *t*-test or Mann–Whitney- or Kruskal–Wallis test for continuous variables, using the same model showed above. We performed multivariable analysis using binomial logistic regression; these analyses were performed only for clinically relevant parameters resulting statistically significant in a univariate analysis. For all conducted analyses *p*-value below 0.05 was considered statistically significant. Analyses were performed using STATA.

## 3. Results

**Figure 1:**
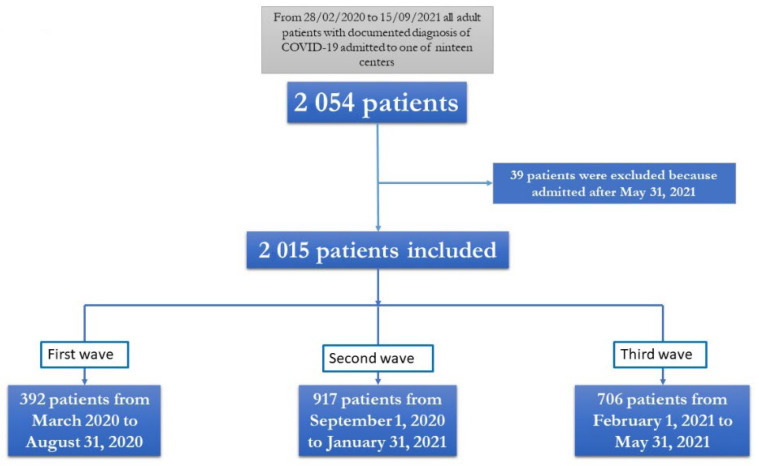
Study flow chart.

Of the 2054 patients included in CoviCamp cohort from 28 February 2020 to 15 September 2021, considering inclusion and exclusion criteria, 2015 patients were included in this study (Figure 1).

Two thousand and fifteen COVID-19 patients were hospitalized during the three waves of the SARS-CoV-2 epidemic in Campania in one of the 17 COVID-19 centers and were enrolled in the present study (Table 1). They were predominantly male (61%) with a median age of 63 years (IQR: 51–74). The median days of hospitalization after the onset of symptoms was 7 (IQR: 3–10), and about 23% of enrolled patients were healthcare workers. The median Charlson comorbidity index was 2 (IQR: 1–4), with hypertension as the most prevalent underlying chronic disease. Fever and dyspnea were the most frequent symptoms at enrolment (58% and 60%, respectively). Median PO_2_/FiO_2_ (ratio of partial pressure arterial oxygen and fraction of inspired oxygen) at enrolment was 230 (IQR: 140–319). Of the 2015 patients enrolled 1393 (69%) had a non-severe clinical outcome and 622 (31%) a severe clinical outcome; 215 patients (10.6%) died during hospitalization.

Table 2 describes the demographic, clinical and hematobiochemical characteristics of the patients enrolled according to the three COVID-19 waves: 392 (19%) were enrolled in the first wave, 917 (45%) in the second and 706 (35%) in the third wave. The median age of the patients in the first wave was younger than the patients enrolled in the second and third waves (median age: 60 years, IQR: 47–71 v.s. 64 years, IQR: 52–75 and 63, IQR: 52–74; *p* = 0.001). The mean Charlson comorbidity index was higher in the second wave than in the first and third waves [3, (IQR: 1–5) in the second-wave, 2 (IQR: 0–4) in the first wave and 2 (IQR: 1–4) in the third wave; *p* = 0.0001]. Cardio-vascular diseases, diabetes and chronic kidney diseases were more frequent in the second wave (23% v.s. 31% v.s. 27%, *p* = 0.011; 16% v.s. 23% v.s. 19%, *p* = 0.006; 8% v.s. 11% v.s. 6%, *p* = 0.001, respectively). No differences in the prevalence of hypertension and chronic obstructive pulmonary disease were observed in the three waves (Table 2).

Considering the symptoms, hypo-ageusia and hypo-anosmia were more frequent in the first wave than in the second and third (20% v.s. 4.1% and 3%, *p* = 0.0001 for hypo-ageusia; 16.9% v.s. 4.1% and 1.7%, *p* = 0.0001 for hypo-anosmia)

A non-severe clinical outcome was observed more frequently in the first and third wave than in the second (73%, 65% and 72%, respectively; *p* = 0.003), but the length of hospitalization expressed in days was higher during the first wave [Median (Q1–Q3): 17 (13–25) v.s. 14 (9–21) v.s. 14 (9–19), respectively, *p* = 0.001)] and also mortality during hospitalization was higher in the first wave than in the second and third waves: 16.6% v.s. 11.3% and 6.5%, respectively (*p* = 0.0001).

Tables S1 and S2 in the Supplementary data showed the characteristics of the patients enrolled during the three waves according to the clinical outcome (non-severe and severe, respectively).

Table 3 shows the characteristics of the patients who died during the hospitalization (215 patients;10.6%) grouped by the period of hospitalization. In the first wave, deaths were more frequently in males than in the second and third waves (72% v.s. 56% v.s. 50%, respectively; *p* = 0.034) and the mean age was younger than in the second and third waves [median, (IQR): 77 (66–82) v.s. 81 (72–86) v.s. 84 (75–88), respectively, *p* = 0.001]. The Charlson comorbidity index of patients who died was worse in the second and third waves than in the first wave [median, (IQR): 3–6) v.s., 5 (4–7) and 6 (5–6), respectively, *p* = 0.0001]. However, the prevalence of chronic disease was similar among the deaths in the three waves.

Finally, Table 4 shows the predictors of mortality: being older [OR: 1.107, CI (10.080–1.135); *p* = 0.0001], having a worse Charlson comorbidity index [OR: 1.135, CI (1.022–1.260); *p* = 0.018] and being enrolled during the first-wave [OR: 1.917, CI (1.054–3.485; *p* = 0.033] were predictors of mortality.

## 4. Discussion

This is a large cohort of patients hospitalized for COVID-19 in Campania, a densely populated region in southern Italy, which may be representative of the cases in our region. Regional healthcare authorities centralized patients with COVID-19 in specialized centers where they received the care and assistance they needed, following local and international guidelines in the 17 COVID-19 centers participating in the present study.

In this study, we characterized the three waves evaluating the demographic, clinical and hematobiochemical characteristics of the patients hospitalized for COVID-19. The subjects admitted in the first wave, although younger, with a lower Charlson comorbidity index and a more frequently mild clinical outcome, showed a higher rate of death during hospitalization and a longer hospitalization than those admitted in the second and third waves. Similarly, analyzing the subjects who died in the three different waves, it is interesting to observe that the deaths in the first wave were more frequently males, were younger and had a lower Charlson comorbidity index than those of the second and third waves.

These data were in agreement with those of another interesting Italian study that evaluated the clinical presentation of COVID-19 during the first and second wave [16]: the 28-day mortality rate was 20.0% (95% CI 16.3 to 23.7) in 449 patients hospitalized for COVID-19 during the first wave v.s. 14.2% (95% CI 12.0 to 16.3) in 1023 patients in the second (log-rank test *p* value = 0.03). In another study, the authors divided the pandemic in two periods, from March 2020 to May 2020 and from June 2020 to August 2020 and noted that the 34,191 patients who died in March–May were significantly younger (80.1 ± 10.6 v.s. 82.8 ± 11.1 years, *p* < 0.001) and less frequently female (41.9% v.s. 61.8%, *p* < 0.001) than the 1104 who died in June–August. Moreover, similar to our results, the patients who died in March–May 2020, compared to those who died in June–August 2020, had significantly lower rates of multiple comorbidities (3 or more comorbidities: 61.8% v.s. 74.5%, *p* = 0.001) [17]. In a Swiss cohort including 930 patients with confirmed SARS-CoV-2 infection hospitalized from 27 February 2020 to 10 May 2021, the crude in-hospital mortality was similar over the course of the first two waves (9.5% and 10.2%, respectively), whereas it decreased in the third wave (5.4%) [18]. Different were the results of Bociąga-Jasik et al. [19] showing in Poland an in-hospital death rate of 10.4% (*n* = 91/875), 19.8% (*n* = 503/2545), and 20.3% (*n* = 359/1771) for waves 1, 2, and 3, respectively (*p* < 0.001), probably because in Poland during the first wave all patients with SARS-CoV-2 infection were supervised by the epidemiological services and their hospitalization was mandatory; so many patients hospitalized during the first pandemic wave were admitted in a good general condition. Then, a Brazilian cohort showed that the mean time between diagnosis and death was 18.5 days in the first wave, 20.5 days in the second wave, and 21.4 days in the third [20]. Another study performed in Lombardy, Italy [21] showed that mortality was significantly higher during the 1st wave than in the following periods (24.2% v.s. 11%; *p* < 0.001).

Thus, most studies showed a less mortality in the second and third waves than in the first wave. This is probably due to the fact that during the first wave, the knowledge on the clinical and therapeutic management of COVID-19 was scanty since it was a completely new disease up to that point. For example, Palmieri et al. [19] showed that treatment patterns were different in the two periods of study: At the beginning of the pandemic the patients who died in March–May 2020 were less likely to be treated with steroids (41.7% v.s. 69.3%, *p* < 0.001) and more likely to receive antivirals (59.3% v.s. 41.4%, *p* < 0.001) than the patients who died in June–August 2020.

In our study, in the first wave, patients were treated with hydroxychloroquine, lopinavir/ritonavir and low-molecular weight heparin (LMWH) at a prophylactic dose, while in the second wave, protease inhibitors and hydroxychloroquine were not used. During second and third waves, LMWH was considered at a prophylactic or therapeutic dosage for patients with severe–critical COVID-19, depending on the clinical judgment or based on ongoing randomized clinical trials [22].

Remdesivir, during the first wave, was available only for compassionate use for patients in an intensive care unit, while during the second and third waves it was routinely prescribed [23].

Corticosteroids, which were not routinely administered during the first wave outside the intensive care unit, were universally prescribed during the second and third waves [24].

As regards tocilizumab, it was limited to small clinical number in the first wave, while its use was intensified in the second and third waves due to the results of the Recovery trial [25].

Between the first, second and third waves, the approach to oxygen therapy also changed. During the first wave, high-flow nasal oxygen (HFNO) and non-invasive ventilation (NIV) via oro-nasal face mask or helmet interface were used only in critical care areas [26], while during the second and third waves these approaches were also used in the COVID-19 wards that did not have an intensive care unit [27,28].

Moreover, it is also important to underscore that the Campania region during the first wave was less affected by COVID-19 than the regions of northern Italy, in particular Lombardy [29]. In fact, during the first wave a total of 100,298 cases were recorded in Lombardy with a cumulative incidence of 992.66 per 100,000; in the same period 6712 cases were recorded in Campania, with a cumulative incidence of 118.01 per 100,000 [30]. Thus, it is clear that during the first wave Campania was able to improve the healthcare organization and to deal better with the subsequent waves, which allowed us to improve our preparedness, health system management and reduce hospital overload. In fact, it was one of the causes that increased the deaths during the pandemic [31,32]. In the Campania Region, beds have been increased and specific clinical units have been created for the treatment of COVID-19, called “COVID-19 centers”, not present before, to take on the pandemic. The health care body was also strengthened, hiring new pulmonologists, cardiologists, intensive care physicians, infectious practitioners and hiring many nurses, setting up an extraordinary organization to deal with the large number of patients with COVID-19 to be managed in hospitals.

Our study shows some limits: first, the retrospective nature of the study; second, we evaluated only hospitalized patients and hospital mortality; third, the lack of some data on the therapy management of the patients; fourth, the absence of analysis of the impact of viral variants. However, we underline that the demographic and clinical characteristics of the patients were like those not included in the present study. The strengths of our study are the multicenter nature of the design and the size of the population.

## 5. Conclusions

In conclusion, the present paper clearly showed that the subjects observed in Campania in the first wave, although younger and with a lower Charlson comorbidity index had a higher rate of death during hospitalization and a longer hospitalization than those admitted in the second and third waves. Thus, it is clear how the improved organization of the healthcare facilities and the increase in the knowledge on clinical and therapeutic management has contributed to the reduction in mortality during the three waves of COVID-19. The knowledge on the clinical management and therapeutic approach gradually improved with a multidisciplinary approach, which saw the collaboration of infectious disease specialists, pulmonologists, cardiologists and intensive care practitioners. We can say that this pandemic has allowed us to directly verify how the study, research and experience can impact the course of a pandemic such as that of COVID-19, which can undoubtedly be defined as epochal.

## Figures and Tables

**Table 1:** Demographic, clinical and hematobiochemical data of patients included in the study.

	Numbers of Data Available
DEMOGRAPHIC VARIABLES	
N° (%) of males	1241 (61.3)
Age, years, Median(Q1–Q3)	63 (51–74)
Days of enrolment after onset of symptoms, Median (Q1–Q3)	7 (3–10)
N° (%) of healthcare workers	23 (1.4)
**CLINICAL AND HEMATOBIOCHEMICAL VARIABLES**	
Charlson comorbidity index, Median (Q1–Q3)	2 (1–4)
**N° (%) of subjects with underlying chronic disease**	
With hypertension	935 (46.2)
With cardio-vascular disease	561 (27.7)
With diabetes	406 (20)
With malignancy	143 (7.1)
With chronic kidney disease	173 (8.5)
With chronic obstructive pulmonary disease	210 (10.4)
With hepatopathy	68 (3.4)
N° (%) of asymptomatic subjects	239 (11.8)
N° (%) of symptomatic patients	1752 (86.5)
N° (%) of symptomatic subjects with	
Fever	1170 (57.8)
Cough	681 (33.6)
Dyspnea	1232 (60.8)
hypo ageusia	108 (5.3)
hypo-anosmia	92 (4.5)
diarrhea	90 (4.4)
cutaneous lesions	10 (0.5)
WBC, Median (Q1–Q3)	7910 (5680–10780)
INR, Median (Q1–Q3)	1.1 (1.02–1.19)
AST, Median (Q1–Q3)	31 (21.7–47)
ALT, Median (Q1–Q3)	31 (20–53)
LDH, Median (Q1–Q3)	300 (234–414)
Creatinine, Median (Q1–Q3)	0.9 (0.7–1.1)
Total bilirubin, Median (Q1–Q3)	0.6 (0.41–0.83)
PO_2_/FiO_2_, Median (Q1–Q3)	230 (140–319)
**OUTCOME**	
**Clinical outcome of COVID-19, n° (%)**	
non-severe COVID-19	1393 (68.8)
severe COVID-19	622 (30.7)
Length of hospitalization expressed in days, median (Q1–Q3)	14 (9–21)
Number of patients who died during hospitalization, N° (%)	215 (10.6)

**Table 2:** Demographic, clinical and hematobiochemical data of patients included, divided by period of hospitalization.

DEMOGRAPHIC VARIABLES	First Wave	Second Wave	Third Wave	*p* Value
**N° of subjects**	**392**	**917**	**706**	
**N° (%) of males**	249 (63.5)	561 (61.2)	431 (61.0)	0.680 ^a^
**Age, years, Median (Q1–Q3)**	60 (47–71)	64 (52–75)	63 (52–74)	0.001 ^b^
**Days of enrolment after onset of symptoms, Median (Q1–Q3)**	5 (3–8)	6 (2–10)	8 (4–10)	0.001 ^b^
**CLINICAL AND HEMATOBIOCHEMICAL VARIABLES**				
**Charlson comorbidity index, Median (Q1–Q3)**	2 (0–4)	3 (1–5)	2 (1–4)	0.001 ^c^
**N° (%) of subjects with underlying chronic disease**				
**With hypertension**	163 (42)	420 (46.2)	352 (49.9)	0.039 ^a^
**With cardio-vascular disease**	89 (22.9)	281 (30.9)	191 (27.01)	0.011 ^a^
**With diabetes**	63 (16.2)	212 (23.3)	131 (18.6)	0.006 ^a^
**With malignancy**	36 (9.3)	68 (7.5)	39 (5.5)	0.061 ^a^
**With chronic kidney disease**	32 (8.2)	100 (11)	41 (5.8)	0.001 ^a^
**With chronic obstructive pulmonary disease**	56 (14.4)	98 (10.8)	56 (8)	0.003 ^a^
**With hepatopathy**	8 (2.1)	31 (3.4)	29 (4.1)	0.196 ^a^
**N° (%) of asymptomatic subjects**	31 (8.4)	149 (16.2)	59 (8.4)	0.001 ^a^
**N° (%) of symptomatic patients**	337 (91.6)	768 (83.8)	647 (91.6)	
**N° (%) OF SYMPTOMATIC SUBJECTS WITH**				
**fever**	237 (70.5)	511 (56.2)	422 (60)	0.001 ^a^
**cough**	141 (41.8)	307 (33.8)	233 (33.2)	0.015 ^a^
**dyspnea**	140 (41.4)	558 (61.4)	534 (76.1)	0.001 ^a^
**hypo ageusia**	50 (20)	37 (4.1)	21 (3.0)	0.001 ^a^
**hypo-anosmia**	43 (16.9)	37 (4.1)	12 (1.7)	0.001 ^a^
**diarrhea**	24 (8.6)	41 (4.5)	25 (3.6)	0.003 ^a^
**cutaneous lesions**	3 (1.5)	3 (0.3)	4 (0.6)	0.144 ^a^
**WBC, Median (Q1–Q3)**	6660 (4010–9210)	8285 (5900–11,090)	7525 (5420–10,400)	0.001 ^c^
**INR, Median (Q1–Q3)**	1.16 (1.06–1.29)	1.09 (1.01–1.2)	1.1 (1.03–1.18)	0.113 ^c^
**AST, Median (Q1–Q3)**	19 (16–27)	30 (20–44)	34 (24–49)	0.001 ^c^
**ALT, Median (Q1–Q3)**	23 (13–37.5)	28 (20–52)	34 (22–57)	0.004 ^c^
**LDH, Median (Q1–Q3)**	198.5 (151–308)	301 (232–419)	301.5 (239–411)	0.008 ^c^
**Creatinine, Median (Q1–Q3)**	0.8 (0.7–1.01)	0.88 (0.7–1.14)	0.9 (0.71–1.09)	0.835 ^c^
**Total bilirubin, Median (Q1–Q3)**	0.59 (0.4–1.13)	0.6 (0.4–0.85)	0.58 (0.41–0.8)	0.811 ^c^
**PO_2_/FiO_2_, Median (Q1–Q3)**	257.5 (119–363)	240 (147–328)	214.5 (133–305)	0.027 ^c^
**OUTCOME**				
**Length of hospitalization expressed in days, median (Q1–Q3)**	17 (13–25)	14 (9–21)	14 (9–19)	0.001 ^c^
**Clinical outcome of COVID-19, N° (%)**				
**non-severe COVID-19**	287 (73.2)	599 (65.3)	503 (71.8)	0.003 ^a^
**severe COVID-19**	105 (26.8)	318 (34.7)	199 (28.2)	0.003 ^a^
**Number of patients who died during hospitalization, N° (%)**	65 (16.6)	104 (11.3)	46 (6.5)	0.001 ^a^

^a^, Chi- square test; ^b^, one-way ANOVA; ^c^, Kruskal-Wallis test.

**Table 3:** Demographic, clinical and hematobiochemical data of patients who died during hospitalization, divided by the period of hospitalization.

	Patients Death in First Wave	Patients Death in Second Wave	Patients Death in Third Wave	*p* Value
**N° of subjects**	**65**	**104**	**46**	
**N° (%) of males**	47 (72.3)	58 (55.8)	23 (50)	0.034 ^a^
**Age, years, mean (SD)**	77 (66–82)	81 (72–86)	84 (75–88)	0.001 ^b^
**Days of enrolment after onset of symptoms, median (Q1–Q3)**	5 (2.5–7)	3 (0–7)	3 (0–8)	0.090 ^b^
**Charlson comorbidity index, median (Q1–Q3)**	3 (2–6)	5 (4–7)	6 (5–6)	0.001 ^c^
**N° (%) of subjects with underlying chronic disease**				
**With hypertension**	34 (54.8)	59 (57.3)	26 (56.5)	0.892 ^a^
**With cardio-vascular disease**	30 (48.4)	53 (51.5)	26 (56.5)	0.704 ^a^
**With diabetes**	16 (25.8)	37 (35.9)	16 (34.8)	0.384 ^a^
**With malignancy**	14 (22.6)	13 (12.6)	5 (10.9)	0.148 ^a^
**With chronic kidney disease**	9 (14.5)	25 (24.3)	10 (21.7)	0.323 ^a^
**With chronic obstructive pulmonary disease**	14 (22.6)	23 (22.3)	6 (13)	0.377 ^a^
**With hepatopathy**	2 (3.2)	8 (7.8)	0 (0)	0.093 ^a^
**N° (%) of asymptomatic subjects**	1 (1.6)	16 (15.4)	4 (8.7)	0.014 ^a^
**N° (%) of symptomatic patients**	62 (98.4)	88 (84.6)	42 (91.3)	
**N° (%) of symptomatic subjects with**				
**fever**	17 (70.8)	42 (40.8)	24 (53.3)	0.022 ^a^
**cough**	10 (41.7)	20 (19.6)	9 (20)	0.060 ^a^
**dyspnea**	11 (45.8)	76 (74.5)	36 (80)	0.007 ^a^
**hypo ageusia**	0 (0)	1 (1.0)	0 (0)	0.739 ^a^
**hypo-anosmia**	0 (0)	1 (1)	0 (0)	0.739 ^a^
**diarrhea**	3 (14.3)	1 (1.0)	0 (0)	0.001 ^a^
**cutaneous lesions**	0 (0)	1 (1)	0 (0)	0.743 ^a^
**Length of hospitalization expressed in days, median (Q1–Q3)**	6 (2–22)	10 (5–15)	8 (4–12)	0.150 ^b^

^a^, Chi- square test; ^b^, one-way ANOVA; ^c^, Kruskal-Wallis test.

**Table 4:** Independent predictors of mortality at multivariable logistic regression analysis.

	OR	95% Lower Confident Interval	95% Lower Confident Interval	*p* Value
Age, years *	1.107	10.080	1.135	0.001
Charlson comorbidity index *	1.135	1.022	1.260	0.018
**Third wave reference value**				
**second wave**	1.078	0.634	1.834	0.781
**first wave**	1.917	1.054	3.485	0.033
Days of enrolment after onset of symptoms *	0.965	0.921	1.010	0.127

* the variable was included in the analysis as continuous parameter.

## Data Availability

The data presented in this study are available on request from the corresponding author.

## Supplementary Materials

The following supporting information can be downloaded at: https://www.mdpi.com/article/10.3390/ijerph192316003/s1, Table S1: Demographic, Clinical and Laboratory data of patients with non-severe outcome grouped by period of hospitalization, Table S2: Demographic, Clinical and Laboratory data of patients with severe outcome grouped by period of hospitalization.

## References

[1] WHO Geneve Listings of WHO’s Response to COVID-19. https://www.who.int/news/item/29-06-2020-covidtimeline.

[2] Macera M., De Angelis G., Sagnelli C., Coppola N., Vanvitelli COVID-Group (2020). Clinical Presentation of COVID-19: Case Series and Review of the Literature. Int. J. Environ. Res. Public Health.

[3] Zhou F., Yu T., Du R., Fan G., Liu Y., Liu Z., Xiang J., Wang Y., Song B., Gu X. (2020). Clinical course and risk factors for mortality of adult inpatients with COVID-19 in Wuhan, China: A retrospective cohort study. Lancet.

[4] Guan W.J., Ni Z.Y., Hu Y., Liang W.H., Ou C.Q., He J.X., Liu L., Shan H., Lei C.L., Hui D.S.C. (2020). Clinical characteristics of coronavirus disease 2019 in China. N. Engl. J. Med..

[5] Wang D., Hu B., Hu C., Zhu F., Liu X., Zhang J., Wang B., Xiang H., Cheng Z., Xiong Y. (2020). Clinical characteristics of 138 hospitalized patients with 2019 novel coronavirus–infected pneumonia in Wuhan, China. JAMA.

[6] Wu Z., McGoogan J.M. (2020). Characteristics of and Important Lessons from the Coronavirus Disease 2019 (COVID-19) Outbreak in China: Summary of a Report of 72,314 Cases from the Chinese Center for Disease Control and Prevention. JAMA.

[7] Kim G.-U., Kim M.-J., Ra S., Lee J., Bae S., Jung J., Kim S.-H. (2020). Clinical characteristics of asymptomatic and symptomatic patients with mild COVID-19. Clin. Microbiol. Infect..

[8] Monari C., Sagnelli C., Maggi P., Sangiovanni V., Numis F.G., Gentile I., Masullo A., Rescigno C., Calabria G., Megna A.S. (2021). More Severe COVID-19 in Patients with Active Cancer: Results of a Multicenter Cohort Study. Front Oncol..

[9] Pisaturo M., Calò F., Russo A., Camaioni C., Giaccone A., Pinchera B., Gentile I., Simeone F., Iodice A., Maggi P. (2021). Dementia as Risk Factor for Severe Coronavirus Disease 2019: A Case-Control Study. Front. Aging Neurosci..

[10] Huang C., Wang Y., Li X., Ren L., Zhao J., Hu Y., Zhang L., Fan G., Xu J., Gu X. (2020). Clinical features of patients infected with 2019 novel coronavirus in Wuhan, China. Lancet.

[11] Guan W.J., Liang W.H., Zhao Y., Liang H.R., Chen Z.S., Li Y.M., Liu X.Q., Chen R.C., Tang C.L., Wang T. (2020). Comorbidity and its impact on 1590 patients with COVID-19 in China: A nationwide analysis. Eur. Respir. J..

[12] Ssentongo P., Ssentongo A.E., Heilbrunn E.S., Ba D.M., Chinchilli V.M. (2020). Association of car-diovascular disease and 10 other pre-existing comorbidities with COVID-19 mortality: A systematic review and meta-analysis. PLoS ONE.

[13] Du Y., Lv Y., Zha W., Zhou N., Hong X. (2021). Association of body mass index (BMI) with critical COVID-19 and in-hospital mortality: A dose-response meta-analysis. Metabolism.

[14] Mahamat-Saleh Y., Fiolet T., Rebeaud M.E., Mulot M., Guihur A., El Fatouhi D., Laouali N., Peiffer-Smadja N., Aune D., Severi G. (2021). Diabetes, hypertension, body mass index, smoking and COVID-19-related mortality: A systematic review and meta-analysis of observational studies. BMJ Open..

[15] Italy: COVID-19 Patients Hospitalized since the Outbreak 2021. Statista. https://www.statista.com/statistics/1125030/covid-19-patients-hospitalized-since-the-outbreak-italy.

[16] Meschiari M., Cozzi-Lepri A., Tonelli R Modena COVID-19 Working Group (2022). First and second waves among hospitalised patients with COVID-19 with severe pneumonia: A comparison of 28-day mortality over the 1-year pandemic in a tertiary university hospital in Italy. BMJ Open.

[17] Palmieri L., Palmer K., Lo Noce C., Meli P., Giuliano M., Floridia M., Tamburo de Bella M., Piccioli A., Brusaferro S., Onder G. (2021). Differences in the clinical characteristics of COVID-19 patients who died in hospital during different phases of the pandemic: National data from Italy. Aging Clin. Exp. Res..

[18] Diebold M., Martinez A.E., Adam K.M., Bassetti S., Osthoff M., Kassi E., Steiger J., Pargger H., Siegemund M., Battegay M. (2021). Temporal trends of COVID-19 related in-hospital mortality and demographics in Switzerland—A retrospective single centre cohort study. Swiss Med. Wkly..

[19] Bociąga-Jasik M., Wojciechowska W., Terlecki M., Wizner B., Rajzer M., Garlicki A., Sładek K., Krzanowska K., Wordliczek J., Krzanowski M. (2022). Comparison between COVID 19 outcomes in the first 3 waves of the pandemic: A reference hospital report. Pol. Arch. Intern. Med..

[20] Dell’Antonio L.S., Leite F.M.C., Dell’Antonio C.S.D.S., Souza C.B., Garbin J.R.T., Santos A.P.B.D., Medeiros Junior N.F., Lopes-Júnior L.C. (2022). COVID-19 Mortality in Public Hospitals in a Brazilian State: An Analysis of the Three Waves of the Pandemic. Int. J. Environ. Res. Public Health.

[21] Leidi F., Boari G.E.M., Scarano O., Mangili B., Gorla G., Corbani A., Accordini B., Napoli F., Ghidelli C., Archenti G. (2022). Comparison of the characteristics, morbidity and mortality of COVID-19 between first and second/third wave in a hospital setting in Lombardy: A retrospective cohort study. Intern. Emerg. Med..

[22] Marietta M., Vandelli P., Mighali P., Vicini R., Coluccio V., D’Amico R., COVID-19 HD Study Group (2020). Randomised controlled trial comparing efficacy and safety of high versus low low-molecular weight heparin dosages in hospitalized patients with severe COVID-19 pneumonia and coagulopathy not requiring invasive mechanical ventilation (COVID-19 HD): A structured summary of a study protocol. Trials.

[23] Farmaci Utilizzabili per IL Trattamento DELLA Malattia COVID-19. Agenzia Italiana del Farmaco. https://aifa.gov.it/aggiornamento-sui-farmaci-utilizzabili-per-il-trattamento-dellamalattia-covid19.

[24] Horby P., Lim W.S., Emberson J.R., Mafham M., Bell J.L., Linsell L., Staplin N., Brightling C., Ustianowski A., RECOVERY Collaborative Group (2021). Dexamethasone in hospitalized patients with COVID-19. N. Engl. J. Med..

[25] RECOVERY Collaborative Group (2021). Tocilizumab in patients admitted to hospital with COVID-19 (RECOVERY): A randomised, controlled, open-label, platform trial. Lancet.

[26] Haymet A., Bassi G.L., Fraser J.F. (2020). Airborne spread of SARS-CoV-2 while using high-flow nasal cannula oxygen therapy: Myth or reality?. Intensive Care. Med..

[27] Sartini C., Tresoldi M., Scarpellini P., Tettamanti A., Carcò F., Landoni G., Zangrillo A. (2020). Respiratory parameters in patients with COVID-19 after using noninvasive ventilation in the prone position outside the intensive care unit. JAMA.

[28] Tonelli R., Pisani L., Tabbì L., Comellini V., Prediletto I., Fantini R., Marchioni A., Andrisani D., Gozzi F., Bruzzi G. (2021). Early awake proning in critical and severe COVID-19 patients undergoing noninvasive respiratory support: A retrospective multicenter cohort study. Pulmonology.

[29] Calò F., Di Fraia A., Russo A., Di Biase A., Misso S., Coppola N. (2022). Blood donor serological screening for SARS-CoV-2 as a tool to estimate the prevalence of asymptomatic infection in a low-intermediate endemic area of southern Italy after the first wave of the pandemic. Blood Transfus..

[30] (2022). Ministry of Health, Rome, Italy. https://www.salute.gov.it/portale/nuovocoronavirus/homeNuovoCoronavirus.jsp.

[31] Kokudo N., Sugiyama H. (2021). Hospital capacity during the COVID-19 pandemic. Glob. Health Med..

[32] Chang A.Y., Cullen M.R., Harrington R.A., Barry M. (2021). The impact of novel coronavirus COVID-19 on noncommunicable disease patients and health systems: A review. J. Intern. Med..

