# Supplementary material for: Clinical Characterization of the Three Waves of COVID-19 Occurring in Southern Italy: Results of a Multicenter Cohort Study
Source: Int J Environ Res Public Health. 2022 Nov 30;19(23):16003. doi: 10.3390/ijerph192316003 (PMC9738780; doi:10.3390/ijerph192316003)
Supplement: Supplementary file 1 [file ijerph-19-16003-s001.zip › ijerph-1983050-supplementary.pdf]

Supplementary Data

Table S1. Demographic, Clinical and Laboratory data of patients with non-severe outcome grouped by period of hospitalization.

	Non-severe COVID 19 First wave	Non-severe COVID 19 Second wave	Non-severe COVID 19 Third wave	P value
**N° of subjects**	**287**	**599**	**503**	
**N° (%) of males**	175(61)	362(60,4)	311(61.3)	0.953[a]
**Age, years, median (Q1-Q3)**	57(45-66)	62(49-72)	60(51-71)	0.0001[b]
**Days of enrolment after onset of symptoms, median (Q1-Q3)**	5(3-8)	6(2-10)	8(5-10)	0.0001[b]
**Charlson comorbidity index, median (Q1-Q3)**	2(0-3)	3(1-4)	2(1-4)	0.0001 [c]
**N° (%) of subjects with underlying chronic disease**				
- **With hypertension**	108(37.8)	263(44.3)	239(47.2)	0.35[a]
- **With cardio-vascular disease**	49(17.1)	167(28.1)	120(23.7)	0.002 [a]
- **With diabetes**	39(13.6)	125(21)	90(17.8)	0.027 [a]
- **With malignancy**	17(5.9)	42(7.1)	27(5.3)	0.483 [a]
- **With chronic kidney disease**	19(6.6)	64(10.8)	22(4.4)	0.0001 [a]
- **With chronic obstructive pulmonary disease**	37(12.9)	50(8.4)	37(7.3)	0.024 [a]
- **With hepatopathy**	5(1.8)	20(3.4)	25(5)	0.059 [a]
**N° (%) of asymptomatic subjects**	28(10.4)	120(20)	53(10.5)	0.0001 [a]
**N° (%) of symptomatic patients**	240(89.6)	479(80)	454(89.5)	
**N° (%) of symptomatic subjects with**				
- **fever**	189(68.7)	325(54.8)	305(60.4)	0.0001 [a]
- **cough**	109(39.5)	193(32.6)	161(32)	0.078 [a]
- **dyspnea**	103(37.1)	317(53.5)	356(70.6)	0.0001 [a]
- **hypo ageusia**	46(21.5)	27(4.6)	15(3)	0.0001 [a]
- **hypo-anosmia**	42(19.3)	29(4.9)	10(2.0)	0.0001 [a]
- **diarrhea**	19(8.1)	30(5.1)	22(4.4)	0.100 [a]
- **cutaneous lesions**	3(1.7)	2(0.3)	3(0.6)	0.138 [a]
**Length of hospitalization expressed in days, median (Q1-Q3)**	17(13-25)	13(8.5-20)	14(9.5-19)	0.0001[c]

a, Chi-square test; b, non-way ANOVA; c, Kruskal-Wallis test.

Table S2. Demographic, Clinical and Laboratory data of patients with severe outcome grouped by period of hospitalization.

	severe COVID 19 First wave	severe COVID 19 Second wave	severe COVID 19 Third wave	P value
**N° of subjects**	**105**	**318**	**199**	
**N° (%) of males**	74(70.5)	199(62.6)	120(60.3)	0.206[a]
**Age, years, median (Q1-Q3)**	73(58-80)	68(57-80)	70(58-81)	0.602[b]
**Days of enrolment after onset of symptoms, Median (Q1-Q3)**	6(3-9.5)	6(2-9)	6.5(3-10)	0.760[b]
**Charlson comorbidity index, median (Q1-Q3)**	3(1-5)	4(2-5)	3(1-5)	0.070[c]
**N° (%) of subjects with underlying chronic disease**				
- **With hypertension**	55(53.9)	157(49.8)	113(56.8)	0.297[a]
- **With cardio-vascular disease**	40(39.2)	114(36.2)	71(35.7)	0.821[a]
- **With diabetes**	24(23.5)	87(27.6)	41(20.6)	0.190[a]
- **With malignancy**	19(18.6)	26(8.3)	12(6)	0.001[a]
- **With chronic kidney disease**	13(12.7)	36(11.5)	19(9.5)	0.667[a]
- **With chronic obstructive pulmonary disease**	19(18.6)	48/15.2)	19(9.5)	0.064[a]
- **With hepatopathy**	3(2.9)	11(3.5)	4(2.0)	0.616[a]
**N° (%) of asymptomatic subjects**	3(3)	29(9.1)	6(3.0)	0.007[a]
**N° (%) of symptomatic patients**	97(97)	289(90.9)	193(97)	
**N° (%) of symptomatic subjects with**				
- **fever**	48(78.7)	186(58.7)	117(59.1)	0.011[a]
- **cough**	32(52.5)	114(36.2)	72(36.4)	0.048[a]
- **dyspnea**	37(61.7)	241(178)	178(89.9)	0.000[a]
- **hypo ageusia**	4(11.1)	10(3.2)	6(3.0)	0.046[a]
- **hypo-anosmia**	1(2.8)	8(2.5)	2(1)	0.459[a]
- **diarrhea**	5(11.1)	11(3.5)	3(1.5)	0.006[a]
- **cutaneous lesions**	0(0)	1(0.3)	1(0.5)	0.894[a]
**Length of hospitalization expressed in days, median (Q1-Q3)**	18(10-28)	16(10-24)	14(8-20)	0.006[c]
**Number of patients who died during hospitalization, N°(%)**	65(61.9)	104(32.7)	46(23.1)	0.000[a]

a, Chi- square test; b, non-way ANOVA; c, Kruskal-Wallis test.
